# Comparative Effectiveness of Different Forms of Telemedicine for Individuals with Heart Failure (HF): A Systematic Review and Network Meta-Analysis

**DOI:** 10.1371/journal.pone.0118681

**Published:** 2015-02-25

**Authors:** Ahmed Kotb, Chris Cameron, Shuching Hsieh, George Wells

**Affiliations:** 1 University of Ottawa Heart Institute, Ottawa, Canada; 2 Department of Community Medicine and Epidemiology, University of Ottawa, Ottawa, Canada; 3 The Ottawa Hospital Research Institute, University of Ottawa, Canada; Providence VA Medical Center and Brown University, UNITED STATES

## Abstract

**Background:**

Previous studies on telemedicine have either focused on its role in the management of chronic diseases in general or examined its effectiveness in comparison to standard post-discharge care. Little has been done to determine the comparative impact of different telemedicine options for a specific population such as individuals with heart failure (HF).

**Methods and Findings:**

Systematic reviews (SR) of randomized controlled trials (RCTs) that examined telephone support, telemonitoring, video monitoring or electrocardiographic monitoring for HF patients were identified using a comprehensive search of the following databases: MEDLINE, EMBASE, CINAHL and The Cochrane Library. Studies were included if they reported the primary outcome of mortality or any of the following secondary outcomes: all-cause hospitalization and heart failure hospitalization. Thirty RCTs (N = 10,193 patients) were included. Compared to usual care, structured telephone support was found to reduce the odds of mortality(Odds Ratio 0.80; 95% Credible Intervals [0.66 to 0.96]) and hospitalizations due to heart failure (0.69; [0.56 to 0.85]). Telemonitoring was also found to reduce the odds of mortality(0.53; [0.36 to 0.80]) and reduce hospitalizations related to heart failure (0.64; [0.39 to 0.95]) compared to usual post-discharge care. Interventions that involved ECG monitoring also reduced the odds of hospitalization due to heart failure (0.71; [0.52 to 0.98]).

**Limitations:**

Much of the evidence currently available has focused on the comparing either telephone support or telemonitoring with usual care. This has therefore limited our current understanding of how some of the less common forms of telemedicine compare to one another.

**Conclusions:**

Compared to usual care, structured telephone support and telemonitoring significantly reduced the odds of deaths and hospitalization due to heart failure. Despite being the most widely studied forms of telemedicine, little has been done to directly compare these two interventions against one another. Further research into their comparative cost-effectiveness is also warranted.

## Introduction

Heart Failure (HF) is a complex debilitating syndrome that results from a cardiac dysfunction that impairs the ability of the ventricle to fill with or eject blood. Living with this disease, individuals with HF experience a substantially high rate of deaths and further cardiac illness. This has led many of those who care for HF patients to believe that they may benefit from more frequent monitoring and follow-up than would otherwise be possible under the current standard of care. This has, in turn, led to the introduction of telemedicine as a potential means for reducing the likelihood of worsening cardiac illness or the prospect of repeated and lengthy hospital readmissions.

Earlier research comparing usual care to a variety of telemedicine interventions demonstrated that the use of telemedicine significantly reduced all-cause mortality and hospital admissions related to heart failure[1–4]. It was suggested as well that while such interventions will require an initial financial investment, they will likely lead to substantially reduced costs in the long term particularly by reducing the costs associated with readmission and hospital stay[5].

Despite these potential benefits, the evidence has been somewhat limited in that studies generally focused on the impact of very broadly defined multifaceted telemedicine interventions as they compared to usual care only rather than on how these inherently different technologies compared to one another. This inconsistency may in part be due to the fact that the definition of telemedicine has varied substantially in their intensity, invasiveness and complexity across studies. Earlier findings regarding the impact of telemedicine for the management of HF have also been relatively inconsistent with more recent ones.

In 2013, a systematic review and network meta-analysis found no significant reductions in all-cause mortality or all-cause hospital readmissions associated with interventions of remote monitoring when compared to usual care for recently discharged patients with heart failure[6]. This review sought to examine the impact of four well-defined forms of telemedicine to determine whether remote monitoring strategies can improve outcomes for adults who have been recently discharged (<28 days) following an unplanned admission due to acute heart failure. As such, there review excluded individuals with stable and chronic heart failure. Most of the studies included in this review followed participants for 6 months or less making it difficult to determine whether or not these interventions can have a lasting effect. They also only included studies with a contemporaneous control group and focused on telephone support interventions (delivered between human to human or human to machine) and telemonitoring (delivered 24 hours a day and 7 days a week or during office hours) making it rather likely that other forms of currently available telemedicine interventions were not included.

An overview of reviews and network meta-analysis of the literature were conducted to quantify, summarize and compare the rates of death, hospitalization and hospitalization due to heart failure for individuals with chronic heart failure who received standard care after discharge or other forms of telemedicine. Network meta-analysis, also known as mixed-treatment comparisons meta-analysis or multiple-treatments meta-analysis would allow the integration of data from direct (when treatments are compared within a randomized trial) and indirect comparisons (when treatments are compared between trials by combining results on how effective they are compared with a common comparator treatment) [7–9]. Simultaneously integrating data using this method can result in greater precision when calculating effect estimates.

Unlike previous reviews, this analysis will not only examine the potential impact of telemedicine against usual care, it will also examine the comparative effectiveness of these different interventions against one another. Interventions compared included usual care and the following five forms of telemedicine: Structured telephone support (STS) which involves regular follow-up calls between the health professional and the patient; Telemonitoring systems which involve the transmission of information on symptoms and signs (TM); Telemonitoring systems and regular telephone follow-up combined; Telemedicine systems involving video monitoring (VIDEO); and Telemedicine systems involving electrocardiographic transmissions (ECG).

## Methods

### Data Sources and Searches

Relevant systematic reviews of randomized controlled trials were identified by searching the following databases until December 2012: The Cochrane Library, Medline, Embase and CINAHL. The search strategies are described in S1 File. There were no restrictions on language, publication year, or type of publication. References of included studies and narrative reviews were searched as well.

### Study Selection

Study selection was conducted by two independent reviewers. All abstracts were examined and reviews were included if they considered the impact of telemedicine interventions in adult heart failure patients. Randomized controlled trials from identified reviews were reviewed in full length and included in the network meta-analysis if they satisfied all of the following criteria:

- Prospective enrolment of consecutive patients with objectively confirmed coronary artery disease with symptomatic heart failure (New York Heart Association [NYHA] Class I-IV) characterized by impaired left ventricular function (Left Ventricular Ejection Fraction ≤ 45%).
- Patients randomized to receive telemedicine (telephone support, telemonitoring, telephone support and telemonitoring together, video monitoring or monitoring by ECG) or usual care. The aforementioned telemedicine strategies are briefly described in Table A in S1 File.
- One or more of the primary outcome or secondary outcomes were reported. The primary outcome measure was all-cause mortality. Secondary outcome measures included all-cause hospitalization (defined as an admission to a health care facility for > 24 hours due to any cause) or hospitalization due to heart failure (defined as an admission to a health care facility for > 24 hours due to worsening heart failure).

Studies were excluded if patients met any of the following exclusion criteria:

- Coronary artery disease patients with preserved left ventricular function (left ventricular ejection fraction >45%)
- Had an acute coronary syndrome or coronary artery bypass surgery within 12 weeks
- Have rheumatic heart disease, severe aortic or mitral valvular heart disease
- Have a medical condition likely to limit survival to < 1 year
- Reside in a nursing facility or receive home visits
- Are unable or unwilling to provide informed consent

### Data Extraction and Quality Assessment

Two reviewers (AK and SH) independently assessed the eligibility of the studies identified in the initial search for inclusion in the review and independently extracted the data from papers considered potentially eligible using a standardized data abstraction form. Both reviewers independently assessed the methodological quality of included reviews using the AMSTAR tool [10] and the quality of included randomized controlled trials using the SIGN-50 checklist [11].

### Data Synthesis and Analysis

Bayesian network meta-analyses and direct frequentist pairwise meta-analyses were conducted for all outcomes. For the primary analysis, the frequency data from each trial were used in the network meta-analysis using WinBUGS (MRC Biostatistics Unit, Cambridge, UK)[12,13]. Bayesian network meta-analysis (NMA) using a binomial likelihood model, which allows for the use of multi-arm trials, were conducted. Random effects network meta-analyses with vague priors were assigned for basic parameters were conducted for the analyses. The WinBUGS code used for the Random Effects Model is available in S1 File [14]. Three chains were fit in WinBUGS for each analysis, with 40,000 iterations, and a burn-in of 40,000 iterations. Odds ratios and 95% credible intervals were modelled using Markov chain Monte Carlo methods. We constructed all evidence networks using NodeXL[15].

Assessment of model fit for NMA comprised of assessment of the deviance information criterion (DIC) and the residual deviance in comparison with the number of unconstrained datapoints [16]. Models with smaller DIC were preferred to models with larger DIC. Similarly, the total value for the residual deviance should be lower than the number of unconstrained data points. To ensure convergence was reached, Brooks-Gelman-Rubin plots were assessed [17]. Model convergence is evident when the Gelman-Rubin statistic approaches 1.

A network meta-analysis also requires that studies are sufficiently similar in order to pool their results. We assessed available study and patient characteristics to ensure similarity and to investigate the potential effect of heterogeneity on effect estimates. Inconsistency was assessed by comparing statistics for the deviance and deviance information criterion in fitted consistency and inconsistency models. Additionally, fixed effects models with vague priors were conducted.

When considered appropriate, pair-wise meta-analyses were conducted by combining studies that compared the same interventions using a random-effects model. Heterogeneity was investigated by examining both forest plots and the inconsistency index (I^2^). [18] I^2^ values of less than 25% represented mild heterogeneity, between 25% and 50% represented moderate heterogeneity, and greater than 50% represented considerable heterogeneity. Results having a p-value of less than 0.05 and 95% confidence intervals (CIs) that excluded 1 were considered to be statistically significant. These analyses were carried out using Comprehensive Meta-Analysis and Review Manager of the Cochrane Collaboration. The results from our network meta-analysis were qualitatively compared with direct, frequentist, pairwise estimates.

## Results

**Fig 1:**
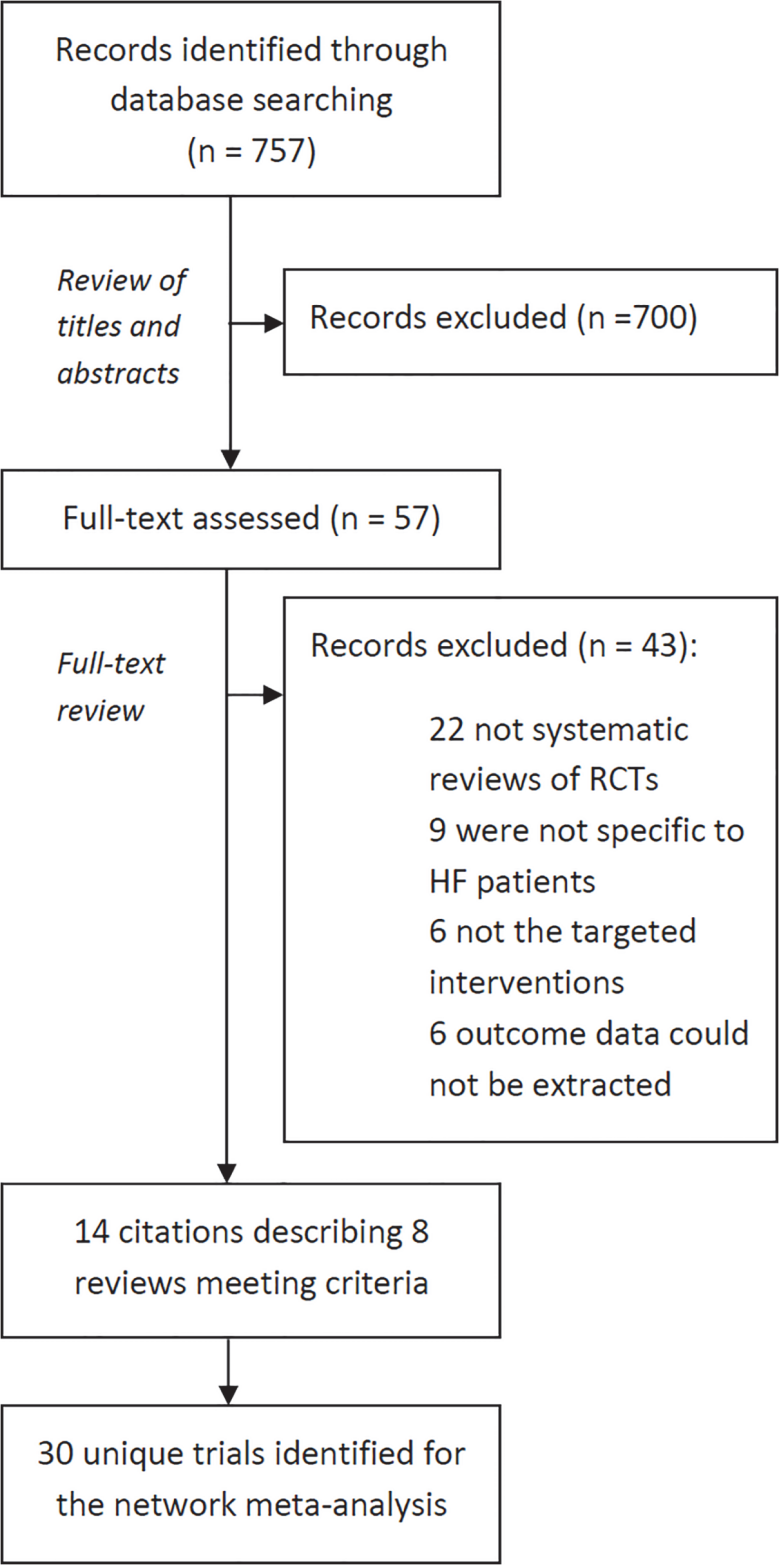
Flow chart for the identification of studies used in the network meta-analysis of telemedicine interventions for heart failure patients

Of the 757 citations identified from our literature search, 700 were excluded after examining their titles and abstracts. The full manuscripts of the remaining 57 were assessed. From those, we identified 8 eligible systematic reviews [1,3,4,18–28]. Fig. 1 shows a modified PRISMA diagram describing the selection of studies. Of the included reviews, 5 included a meta-analysis of the data. Six out of the 8 reviews were found to have met the following criteria: duplicate study selection and data extraction; comprehensive literature search; provided characteristics of their included studies; and assessed their included studies’ scientific quality. Characteristics of these reviews and their AMSTAR quality assessment are detailed in Tables B and C in S1 File.

Thirty eligible randomized controlled trials[29–59], with a total of 10,193 patients, were then identified from the systematic reviews and included in the network meta-analysis. In most randomized controlled trials, a single telemedicine intervention was compared with usual care. For most studies (21 out of 30) patients mean age was greater than 65 and in all but one study the patients were mostly males. In 27 of 30 trials, participants were followed for 6 or more months and in 25 trials the intervention was delivered for 6 or more months. However, the frequency of delivering the intervention did vary considerably. In most trials, the health professional that typically delivered the intervention was a nurse. A more detailed description of included trials is provided in Table 1.

**Table 1:** Description of included studies.

Author/Year	Study population	Interventions	Follow-up lengths	Sign-50
Angermann 2007	Chronic HF Patient age was 68+/−12 years, 29% were female, and 40% were in NYHA class III-IV.	Structured telephone support vs. Usual Care	6 months duration	Acceptable
Balk 2008	Median age was 66 years, 89% had systolic Left Ventricular dysfunction, and 90% were in NYHA class II or III.	Telemonitoring vs. Usual Care	mean follow-up duration of 9.6 months	High quality
Blum 2007	Age 72; 28% female and 46% were NYHA class III.	Telemonitoring including ECG data transmission vs. Usual care	Mean follow-up of 24 months	Acceptable
Capomolla 2004	Chronic HF; age 57; Male:Female ratio was 117/16; NYHA II/III-IV 89/44	Both Structured telephone support and Telemonitoring vs. Usual Care	12 months	High quality
Cleland 2005	48% were aged> 70 years; NYHA class IV heart failure in the previous month, although 62% reported well-controlled symptoms (NYHA functional class I/II)	Telemonitoring including ECG data transmission vs. Structured telephone support vs. Usual care	8 months	High quality
De Lusignan 2001	Chronic HF aged 75, average NYHA 1.75	Video monitoring vs. Usual Care	12 months	Unacceptable
DeBusk 2004	mean age of patients was 72; 51% NYHA III or IV; 51% Male	Structured telephone support vs. Usual Care	12 months	High quality
Dendale 2012	Chronic HF; mean age 76; 65% male;	Telemonitoring vs. Usual Care	6 months	High quality
DeWalt 2006	Mean age 62; 58% male in intervention group and 41% in control; 40% in intervention were NYHA III and 51% in control	Structured telephone support vs. Usual Care	12 months	High quality
Ekman 1998	Mean age 80; 42% females	Structured telephone support vs. Usual Care	6 months	High quality
Galbreath 2004	Mean age 70.9; 29% female; 21% NYHA III	Structured telephone support vs. Usual Care	18 months	High quality
GESICA 2005	The mean age was 65 years, 71% were men, most patients were in New York Heart Association (NYHA) class II or III	Structured telephone support vs. Usual Care	16 months	High quality
Giordano 2009	Chronic HF; Aged 57; 16% female in Telemonitoring group and 14% in usual care; NYHA III-IV 46% in TM and 35% in UC	Telemonitoring vs. Usual Care	12 months	High quality
Goldberg 2003	Mean age was 59 and 68% were male; 75% of NYHA III and 24% in NYHA IV	Telemonitoring vs. Usual Care	6 months	High quality
Kielblock 2007	Chronic HF aged approx. 73, 42.6% female in I and 55.3% in Control	Telemonitoring vs. Usual Care	12 months	Unacceptable
Koehler 2011	Chronic HF; aged 67 n approx. 80% male; 50% NYHA III and 50% II	Telemonitoring including ECG data transmission vs. Usual care	26 months	High quality
Krum 2009	Chronic HF; aged 75; 65% male; 58.2% NYHA III and IV	Structured telephone support vs. Usual Care	12 months	Acceptable
Krumholz 2002	HF and the median age of the patients was 74 years; 57% were men	Structured telephone support vs. Usual Care	12 months	Acceptable
Laramee 2003	Mean age 70; 42% female in the Intervention group and 50% in Control	Structured telephone support vs. Usual Care	3 months	Acceptable
Mortara 2009	Age 60; approx. 15% female	Structured telephone support vs. Both Structured telephone support and Telemonitoring vs. Usual Care	12 months	Acceptable
Ramachandran 2007	Age 44; 22% Female; 74% NYHA I and II	Structured telephone support vs. Usual Care	6 months	Acceptable
Riegel 2002	Age 73, female 46% in the Intervention group and 54% in Control	Structured telephone support vs. Usual Care	6 months	Unacceptable
Riegel 2006	Age 72; 54% female; 81% NYHA III/IV	Structured telephone support vs. Usual Care	6 months	High quality
Schwarz 2008	Age 78; 43% female in the Intervention group and 61% in Usual Care	Telemonitoring to patients and their caregivers vs. Usual Care	3 months	High quality
Sisk 2006	Age 60; 47% females; approx. 45% NYHA IV	Structured telephone support vs. Usual Care	12 months	High quality
Villani 2007	Age 64 in Control group and 69 in the intervention group; 75% male	Telemonitoring including ECG data transmission vs. Usual care	12 months	Acceptable
Wade 2011	Approx. 76 age; 48% female	Telemonitoring + Case Management (CM) (calls/education) vs. CM (calls/education)	6 months	Acceptable
Wakefield 2008	Age 69; 99% male; 65% NYHA III	Video monitoring vs. Telephone support vs. Usual Care	12 months	High quality
Woodend 2008	Age 67; 72% male; approx. 62% NYHA III or higher	Video monitoring vs. Usual Care	12 months	High quality
Zugck 2008	Age 62; 85.5% male; 80% NYHA II	Telemonitoring vs. Usual Care	3 months	Acceptable

Using the SIGN-50 assessment tool, 17 of the 30 randomized controlled trials were judged to be of high quality, 10 were acceptable and 3 were considered of poor quality. Generally, trials were judged to have appropriately randomized patients, adequately concealed allocation, similar groups at baseline, and had few losses to follow-up and analyzed patients according to the intention to treat. A summary of the quality of the trials is provided in Table 1 and further details on the assessment of trials using the SIGN-50 tool are provided in Table D in S1 File.

### Direct comparisons

**Fig 2:**
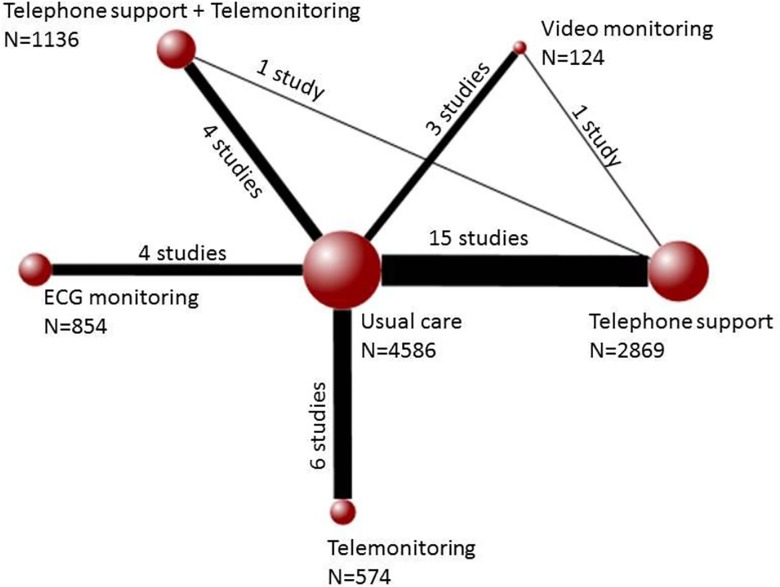
Evidence network for interventions included in the analysis of all-cause mortality. Each node represents an intervention and the size of each node indicates how many patients received it of the total number of patients included in the network (N = 10,193). The solid lines connecting the nodes together indicate the existence of this comparison of interventions in the literature. The thickness of the lines represents how many studies of the total number of studies (30 studies) include a particular comparison.

Twenty-nine trials contributed to the analysis of the outcome of death, twenty to the analysis of hospitalization and sixteen for the analysis of hospitalization due to heart failure. Of the 15 possible pairwise comparisons that can be made across the 6 interventions, the evidence available was found to have only examined 8 comparisons directly for death as well as for hospitalization. Six out of 10 possible pairwise comparisons were available for hospitalization due to heart failure. Fig. 2 shows the evidence network for the outcome of death. For the outcomes of hospitalization and heart failure hospitalization, the evidence networks are provided in Figs. A and B in S1 File.

**Fig 3:**
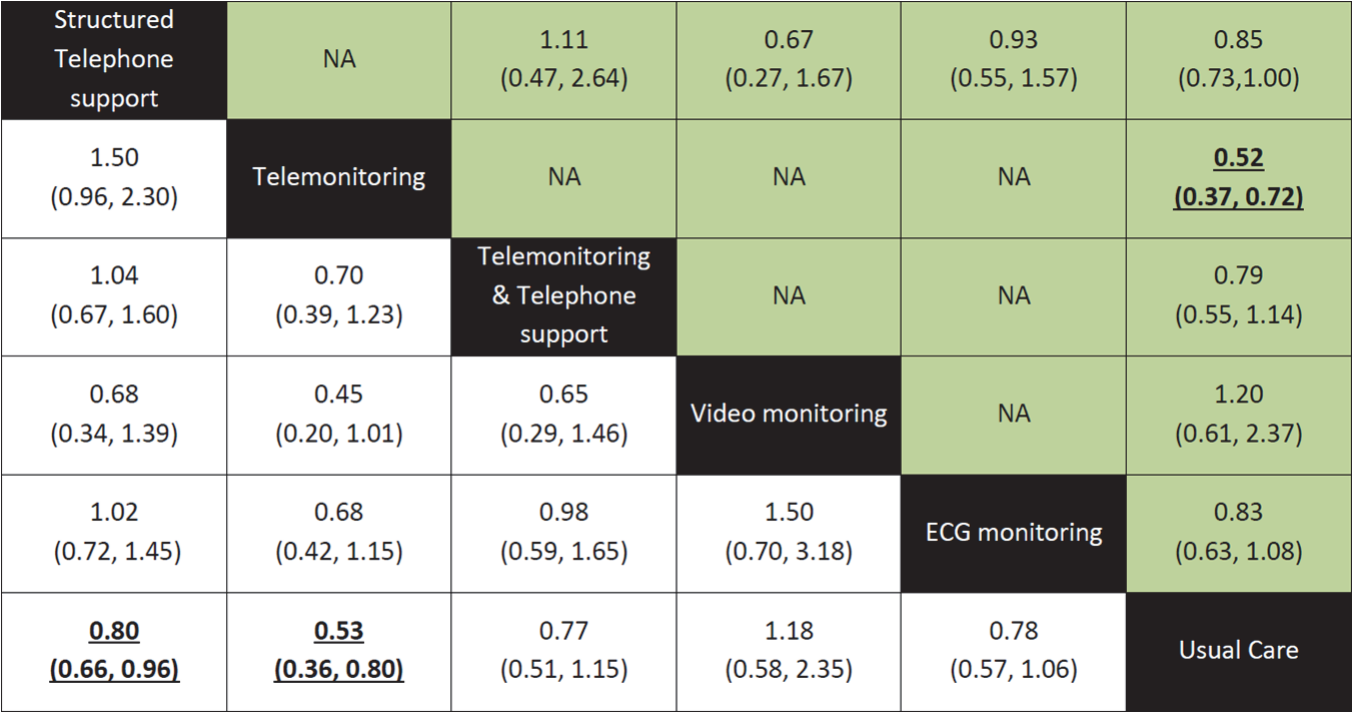
The impact of different forms of telemedicine on the outcome of all-cause mortality. Effect estimates from the network meta-analysis occupy the bottom left part of the diagram, the estimates from the pairwise meta-analyes occupy the top right part of the diagram and the diagonal corresponds to the comparison. The odds ratios and 95% Credible Intervals for the comparisons in this diagram should be read from left to right (e.g. Patients receiving structured telephone support had a 0.80 [0.66, 0.96] reduced odds of death compared to those receiving usual care). Significant results are underlined and in bold.

Direct comparisons (Fig. 3, S1 and S2 Figs.) show that telemonitoring was found to be more effective than usual care in reducing the numbers of death (Odds ratio (OR) 0.52 95% Confidence Intervals (CI) [0.37, 0.72]), hospitalization (0.70 [0.51, 0.96]), and hospitalization resulting from heart failure (0.70 [0.51, 0.98]). Fewer patients receiving structured telephone support interventions were hospitalized for all causes (0.86 [0.77, 0.97)] and due to heart failure (0.76 [0.65, 0.89)] than patients who received usual care. Similarly, fewer patients who received telemedicine interventions that involved the use of ECG data transmission were hospitalized than patients who received usual care (0.70 [0.55, 0.91]). No other comparisons were found to suggest a significant benefit across the outcomes of death, hospitalization and heart failure related hospitalization for one intervention over the other. For all outcomes, heterogeneity was found to be either low or moderate. Forest plots of each pairwise meta-analysis conducted can be found in Fig. C in S1 File.

### Incorporation of Data from the Indirect Comparisons of Interventions

Across all outcomes the Brooks-Gelman-Rubin plots demonstrated model convergence. An assessment of model fit and inconsistency is described in the Tables E-H and Figs. D-F in S1 File.

***All-cause mortality***. Compared with usual care, the only interventions that significantly reduced the odds of death were structured telephone support [OR 0.80 95% Credible Intervals (CrI) (0.66, 0.96)] and telemonitoring [0.53 (0.36, 0.80)]. No other significant differences were observed across treatment comparisons (Fig. 3). In terms of potentially reducing the odds of death, telemonitoring ranked first, followed by structured telephone support delivered in combination with telemonitoring, electrocardiographic data transmission, structured telephone support, usual care and video monitoring.

***All-cause hospitalization***. For the most part, the odds of hospitalization did not significantly vary across interventions (S1 Fig.) making these results relatively consistent with the results from the direct pairwise analyses. The main difference however, was that in this analysis, both structured telephone support and telemonitoring were no longer found to significantly reduce all-cause hospitalization compared to usual care. However, these results are considered more robust given their incorporation of all available data from both direct and indirect comparisons. According to their relative potential for reducing hospitalizations, telemonitoring was ranked first, followed by video monitoring, structured telephone support, electrocardiographic data transmission, usual care and structured telephone support and telemonitoring.

***Heart failure hospitalization***. The incorporation of indirect evidence confirmed that structured telephone support interventions [OR 0.69 95% CrI (0.56, 0.85)], telemonitoring interventions [0.64 (0.39, 0.95)] and telemedicine that included the transmission of electrocardiographic data [0.71 (0.52, 0.98)] all significantly reduced hospitalizations due to heart failure compared to usual care. The remaining comparisons did not show favor for one intervention over the other (S2 Fig.). Once again, telemonitoring interventions ranked first, followed by structured telephone support, telemedicine that involved electrocardiographic data transmission, structured telephone support and telemonitoring interventions delivered together and usual care.

***Sensitivity analyses***. To examine other potential sources of heterogeneity in the network, the following areas were examined across all included studies in subsequent sensitivity analyses: randomization, concealment of allocation, degree of loss to follow-up, and the inclusion of all randomized participants in the analysis according to the intention to treat principle. Additional sensitivity analyses were conducted by repeating the primary analysis using a fixed-effect method. The results of these analyses are in Tables I-N in S1 File.

## Discussion

Telemonitoring as well as structured telephone support interventions were both found to be significantly better than usual care in reducing deaths and heart failure related hospitalizations. Telemedicine interventions that involved the use of electrocardiographic (ECG) data transmission were also significantly more effective in reducing hospitalizations due to heart failure when compared with usual care. There were no other significant differences found across the interventions compared. This review and network meta-analysis adheres to PRISMA reporting standards (S1 PRISMA Checklist) and is also the only one to date that compares the aforementioned forms of telemedicine against one another as well as standard post-discharge care. The advantage of conducting this multiple treatment comparison (MTC) meta-analysis was that it made possible the incorporation data from both indirect and direct comparisons.

Most of the evidence that is currently available on the impact of telemedicine interventions involves the comparison of an active form of telemedicine to standard care. As such, findings from this network meta-analysis are unique in that they examine the various comparisons across five main forms of active telemedicine interventions. For the first time, the currently available interventions were ranked according to their effectiveness in reducing the outcomes of death, hospitalization and hospitalization due to heart failure. When these different interventions were ranked, telemonitoring was always ranked first.

Findings from this review confirm the findings of previous systematic reviews and meta-analysis [1,3,4] and extend beyond them. Previous studies have concentrated on the effectiveness of telemedicine compared to usual care. A recent review by Xiang (2013) demonstrated that interventions such as telemonitoring or nurse administered telephone-based management programs were clinically effective in patients with chronic heart failure when compared with usual care. The estimates obtained from their meta-analysis and meta-regression were very similar for the outcomes of mortality and heart failure-related hospitalization to the outcomes obtained in this network meta-analysis [60]. Additionally, they examined the outcome of heart failure-related hospital length of stay and found that telehealth programs were associated with as significant reduction in this outcome when compared with usual care. However, this meta-analysis is limited in that it does not examine how these various forms of currently available telemedicine interventions perform when compared against one another and not just against usual care.

A recent network meta-analysis of telemedicine interventions for individuals with acute heart failure demonstrated that telephone support delivered from human to human and telemonitoring delivered during office hours showed beneficial trends compared to usual care, particularly in reducing all-cause mortality [6]. Despite both results trending in the same direction, the results described in this previous review, did not reach statistical significance. This difference may suggest that chronic heart failure patients, who are more stable, may stand to benefit more from telemedicine. To definitively determine this, further research is warranted. The difference in results may also be due to the inclusion of a smaller amount of evidence. In this review, 30 studies were included if they compared five forms of telemedicine to usual care or to each other. In the previous network meta-analysis, 21 studies were included if they included a control group and examined the impact of telephone support or telemonitoring. Finally, only 9 out of 21 studies were followed participants for more than 6 months. In this review, 19 out of 30 studies had longer than 6 months of follow-up. This may suggest that the potential benefits of telemedicine require longer periods of follow-up before they are observed.

This review demonstrated that the amount of evidence available in the literature for directly comparing across the active forms of telemedicine was limited. Since much of the currently available evidence has focused more on telephone support and telemonitoring interventions, other widely available forms of telemedicine such as video monitoring and monitoring by ECG remain relatively understudied. Further research is still needed before more definitive conclusions can be made regarding their effectiveness. However, the ability to integrate evidence from direct and indirect comparisons as a result of conducting this network meta-analysis allowed for a gain of statistical precision compared with previous reviews and allowed for the comparison of interventions that had not been previously compared in the literature and. This network analysis was limited to only including randomized controlled trials. This was deemed appropriate, however, given the availability of a substantial amount of evidence and the reduced likelihood of bias and confounding associated with this study design. As is the case with any meta-analysis, the strength of the analysis depends on the quality and the completeness of the available evidence. For the most part, the risk of bias associated with included studies was found to be either low or acceptable and further sensitivity analyses did not significantly differ from the study’s main analysis.

In summary, this analysis has demonstrated that structured telephone support and telemonitoring interventions and may be of significant benefit for rehabilitating heart failure patients. This work represents the first application of network meta-analysis to examine the comparative effectiveness of five telemedicine interventions in improving heart failure patient outcomes. Findings from this network meta-analysis confirm the findings of previous reviews [1,3,4] that telemedicine may significantly improve patient outcomes beyond usual care and extend beyond them by comparing these different forms of telemedicine against each other. Further research is needed to examine the long-term impact and cost-effectiveness of telemonitoring and structured telephone support interventions on specific subsets of heart failure patients considered most likely to benefit.

## Supporting Information

S1 PRISMA Checklist PRISMA Checklist. (DOCX)

S1 Fig The impact of different forms of telemedicine on the outcome of all-cause hospitalization. Effect estimates from the network meta-analysis occupy the bottom left part of the diagram, the estimates from the pairwise meta-analyes occupy the top right part of the diagram and the diagonal corresponds to the comparison. The odds ratios and 95% Credible Intervals for the comparisons in this diagram should be read from left to right (e.g. Patients receiving structured telephone support had a 0.86 [0.77, 0.97] reduced odds of all-cause hospitalization compared to those receiving usual care). Significant results are underlined and in bold.
(TIF)

S2 Fig The impact of different forms of telemedicine on the outcome of hospitalization due to heart failure. Effect estimates from the network meta-analysis occupy the bottom left part of the diagram, the estimates from the pairwise meta-analyes occupy the top right part of the diagram and the diagonal corresponds to the comparison. The odds ratios and 95% Credible Intervals for the comparisons in this diagram should be read from left to right (e.g. Patients receiving structured telephone support had a 0.69 [0.56, 0.85] reduced odds of hospitalization due to heart failure compared to those receiving usual care). Significant results are underlined and in bold.
(TIF)

S1 File Appendix file. (DOCX)
