# Supplementary figures and images for: Comparative Effectiveness of Different Forms of Telemedicine for Individuals with Heart Failure (HF): A Systematic Review and Network Meta-Analysis
Source: PLoS One. 2015 Feb 25;10(2):e0118681. doi: 10.1371/journal.pone.0118681 (PMC4340962; doi:10.1371/journal.pone.0118681)

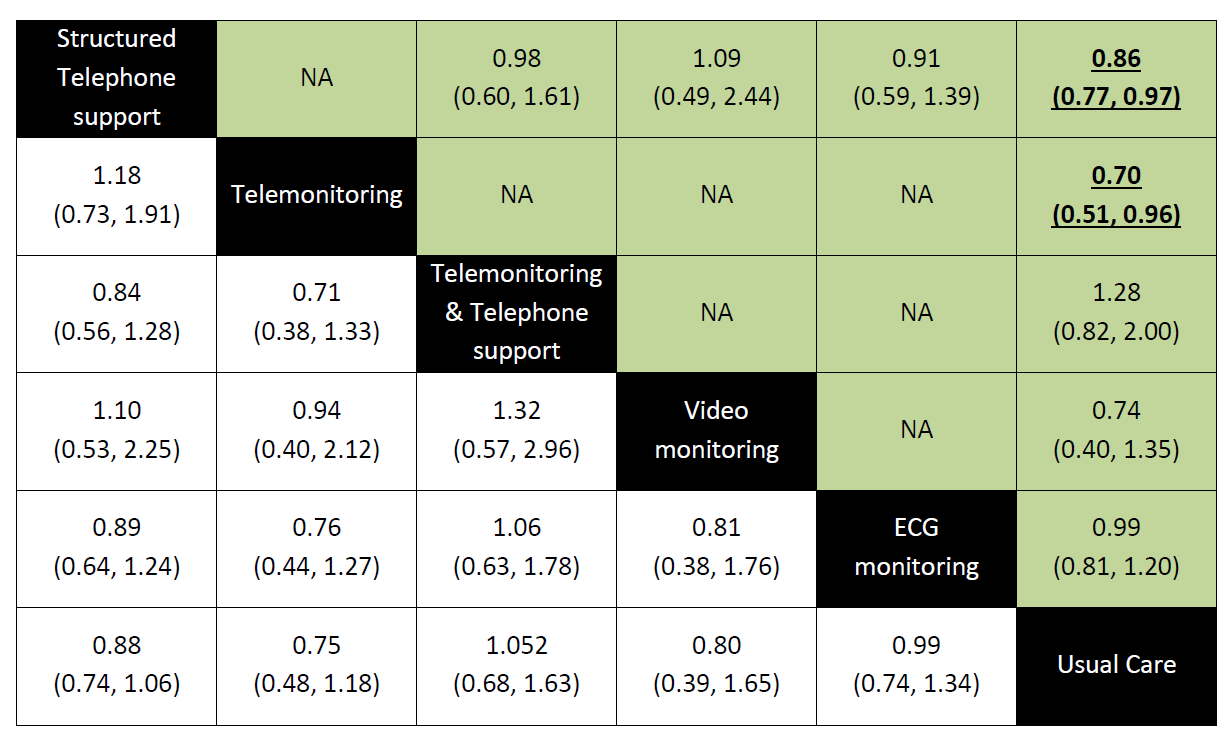

Supplement: S1 Fig — Effect estimates from the network meta-analysis occupy the bottom left part of the diagram, the estimates from the pairwise meta-analyes occupy the top right part of the diagram and the diagonal corresponds to the comparison. The odds ratios and 95% Credible Intervals for the comparisons in this diagram should be read from left to right (e.g. Patients receiving structured telephone support had a 0.86 [0.77, 0.97] reduced odds of all-cause hospitalization compared to those receiving usual care). Significant results are underlined and in bold. (TIF) [file pone.0118681.s002.tif]

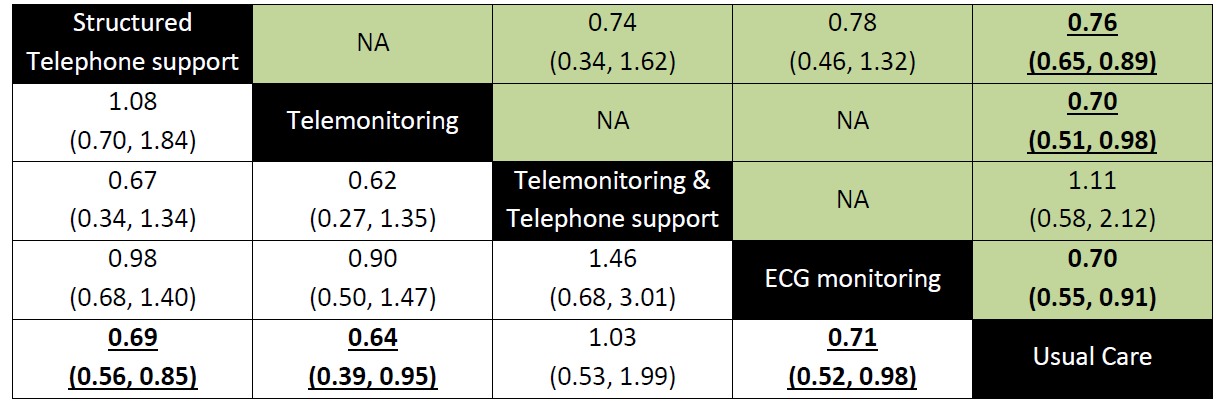

Supplement: S2 Fig — Effect estimates from the network meta-analysis occupy the bottom left part of the diagram, the estimates from the pairwise meta-analyes occupy the top right part of the diagram and the diagonal corresponds to the comparison. The odds ratios and 95% Credible Intervals for the comparisons in this diagram should be read from left to right (e.g. Patients receiving structured telephone support had a 0.69 [0.56, 0.85] reduced odds of hospitalization due to heart failure compared to those receiving usual care). Significant results are underlined and in bold. (TIF) [file pone.0118681.s003.tif]
