# Supplementary material for: Comparative Effectiveness of Different Forms of Telemedicine for Individuals with Heart Failure (HF): A Systematic Review and Network Meta-Analysis
Source: PLoS One. 2015 Feb 25;10(2):e0118681. doi: 10.1371/journal.pone.0118681 (PMC4340962; doi:10.1371/journal.pone.0118681)
Supplement: S1 File — (DOCX) [file pone.0118681.s004.docx]

# Supplementary Appendix

## Electronic search strategy

**Medline**

1 telemedicine.mp. (11504)

2 exp telemedicine/ (14013)

3 telehealth.mp. (1311)

4 exp Telecommunications/ (55750)

5 telecommunication.mp. (1216)

6 reminder system.mp. (264)

7 exp Reminder Systems/ (2002)

8 "home monitor*".ab,ti. (1092)

9 telecardiology.ab,ti. (137)

10 "telemonitor*".ab,ti. (537)

11 "remot* monitor*".mp. (632)

12 telephone support.mp. (314)

13 exp Speech Recognition Software/ (432)

14 Speech Recognition Software.mp. (465)

15 reminder systems.mp. (2160)

16 exp Teleradiology/ (1191)

17 teleradiology.mp. (1492)

18 telecardiology.mp. (137)

19 exp Telemetry/ (7950)

20 telemetry.mp. (9631)

21 interactive voice response/ or interactive voice response.mp. (363)

22 1 or 2 or 3 or 4 or 5 or 6 or 7 or 8 or 9 or 10 or 11 or 12 or 13 or 14 or 15 or 16 or 17 or 18 or 19 or 20 or 21 (63348)

23 exp heart failure/ (80322)

24 heart failure.mp. (125397)

25 exp coronary disease/ (174338)

26 coronary disease.mp. (128972)

27 (coronary adj2 disease).ab,ti. (99577)

28 exp myocardial infarction/ (139962)

29 "myocardial infarction".ab,ti. (122311)

30 CABG.mp. (11686)

31 exp Coronary Artery Bypass/ (42946)

32 coronary artery bypass.mp. (49943)

33 PCI.mp. (11200)

34 PTCA.mp. (6087)

35 exp Angioplasty, Balloon, Coronary/ (32274)

36 exp Coronary Artery Disease/ (33262)

37 coronary artery disease.mp. (74636)

38 "heart attack".ab,ti. (2890)

39 "heart infarction".ab,ti. (191)

40 "cardiac arrest".ab,ti. (18230)

41 exp Acute Coronary Syndrome/ (5862)

42 acute coronary syndrome.mp. (11449)

43 acs.mp. (9357)

44 cardiac failure.mp. (9616)

45 ischemic heart disease.mp. or exp Myocardial Ischemia/ (345055)

46 angina.mp. or exp Angina, Stable/ or exp Angina Pectoris/ or exp Angina, Unstable/ (62861)

47 23 or 24 or 25 or 26 or 27 or 28 or 29 or 30 or 31 or 32 or 33 or 34 or 35 or 36 or 37 or 38 or 39 or 40 or 41 or 42 or 43 or 44 or 45 or 46 (551918)

48 22 and 47 (1822)

49 Meta-Analysis as Topic/ (12681)

50 meta analy$.tw. (48875)

51 metaanaly$.tw. (1214)

52 Meta-Analysis/ (38678)

53 (systematic adj (review$1 or overview$1)).tw. (40894)

54 exp Review Literature as Topic/ (6684)

55 49 or 50 or 51 or 52 or 53 or 54 (98559)

56 cochrane.ab. (24378)

57 embase.ab. (22086)

58 (psychlit or psyclit).ab. (877)

59 (psychinfo or psycinfo).ab. (8479)

60 (cinahl or cinhal).ab. (8143)

61 science citation index.ab. (1760)

62 bids.ab. (339)

63 cancerlit.ab. (568)

64 56 or 57 or 58 or 59 or 60 or 61 or 62 or 63 (40020)

65 reference list$.ab. (8427)

66 bibliograph$.ab. (10669)

67 hand-search$.ab. (3464)

68 relevant journals.ab. (619)

69 manual search$.ab. (2014)

70 65 or 66 or 67 or 68 or 69 (22537)

71 selection criteria.ab. (17698)

72 data extraction.ab. (8577)

73 71 or 72 (24850)

74 Review/ (1770613)

75 73 or 74 (1778932)

76 Comment/ (528661)

77 Letter/ (786831)

78 Editorial/ (323802)

79 animal/ (5114599)

80 human/ (12764478)

81 79 not (79 and 80) (3731794)

82 or/76-78,81 (4911854)

83 55 or 64 or 70 or 75 (1836799)

84 83 not 82 (1676453)

85 48 and 84 (183)

**EMBASE**

1 telemedicine.mp. or exp telemedicine/ or exp telecommunication/ (33029)

2 telecommunication.mp. (16756)

3 reminder systems.mp. or exp reminder system/ (1540)

4 telehealth.mp. or exp telehealth/ (16787)

5 home monitoring.mp. or exp home monitoring/ (3388)

6 "home monitor*".mp. (3488)

7 telecardiology.mp. or exp teleconsultation/ or exp telecardiology/ or exp telemetry/ (19056)

8 exp teleradiology/ or teleradiology.mp. (1523)

9 "remot* monitor*".mp. (1075)

10 "telemonitor*".mp. (1074)

11 telephone support.mp. (442)

12 Speech Recognition Software.mp. or exp automatic speech recognition/ (518)

13 exp interactive voice response system/ or interactive voice response.mp. (550)

14 telemetry.mp. (14066)

15 1 or 2 or 3 or 4 or 5 or 6 or 7 or 8 or 9 or 10 or 11 or 12 or 13 or 14 (55149)

16 heart failure.mp. or exp heart failure/ (307863)

17 coronary disease.mp. or exp coronary artery disease/ (218972)

18 coronary artery disease.mp. (173166)

19 (coronary adj2 disease).ab,ti. (137077)

20 myocardial infarction.mp. or exp heart infarction/ (293858)

21 heart infarction.mp. (254831)

22 exp coronary artery bypass graft/ or CABG.mp. (56812)

23 coronary artery bypass.mp. (67715)

24 exp percutaneous coronary intervention/ or PCI.mp. (60159)

25 percutaneous coronary intervention.mp. or exp transluminal coronary angioplasty/ (54419)

26 exp percutaneous transluminal angioplasty balloon/ or exp angioplasty/ or exp percutaneous transluminal angioplasty/ or angioplasty.mp. (75926)

27 heart attack.mp. (3796)

28 cardiac arrest.mp. or exp heart arrest/ (50240)

29 cardiac failure.mp. or exp heart failure/ (283371)

30 angina.mp. or exp angina pectoris/ (96405)

31 ischemic heart disease.mp. or exp ischemic heart disease/ (470464)

32 acute coronary syndrome.mp. or exp acute coronary syndrome/ (25722)

33 acs.mp. (15431)

34 16 or 17 or 18 or 19 or 20 or 21 or 22 or 23 or 24 or 25 or 26 or 27 or 28 or 29 or 30 or 31 or 32 or 33 (915732)

35 exp Meta Analysis/ (67825)

36 ((meta adj analy$) or metaanalys$).tw. (63259)

37 (systematic adj (review$1 or overview$1)).tw. (48992)

38 35 or 36 or 37 (125392)

39 cancerlit.ab. (667)

40 cochrane.ab. (28842)

41 embase.ab. (25782)

42 (psychlit or psyclit).ab. (957)

43 (psychinfo or psycinfo).ab. (6385)

44 (cinahl or cinhal).ab. (8749)

45 science citation index.ab. (1915)

46 bids.ab. (436)

47 39 or 40 or 41 or 42 or 43 or 44 or 45 or 46 (44036)

48 reference lists.ab. (8639)

49 bibliograph$.ab. (17743)

50 hand-search$.ab. (3980)

51 manual search$.ab. (2283)

52 relevant journals.ab. (724)

53 48 or 49 or 50 or 51 or 52 (30491)

54 data extraction.ab. (10629)

55 selection criteria.ab. (19478)

56 54 or 55 (28750)

57 review.pt. (1919616)

58 56 or 57 (1931280)

59 letter.pt. (807623)

60 editorial.pt. (421593)

61 animal/ (1810429)

62 human/ (14108254)

63 61 not (61 and 62) (1354958)

64 or/59-60,63 (2570545)

65 38 or 47 or 53 or 58 (2015633)

66 65 not 64 (1950005)

67 15 and 34 (2891)

68 66 and 67 (373)

**The Cochrane Library**

1 telemedicine.mp. [mp=title, short title, abstract, full text, keywords, caption text] (46)

2 telehealth.mp. [mp=title, short title, abstract, full text, keywords, caption text] (21)

3 telecommunication.mp. [mp=title, short title, abstract, full text, keywords, caption text] (23)

4 reminder systems.mp. [mp=title, short title, abstract, full text, keywords, caption text] (37)

5 "home monitor*".mp. [mp=title, short title, abstract, full text, keywords, caption text] (16)

6 telecardiology.mp. [mp=title, short title, abstract, full text, keywords, caption text] (1)

7 teleradiology.mp. [mp=title, short title, abstract, full text, keywords, caption text] (3)

8 "remot* monitor*".mp. [mp=title, short title, abstract, full text, keywords, caption text] (4)

9 "telemonitor*".mp. [mp=title, short title, abstract, full text, keywords, caption text] (8)

10 telephone support.mp. [mp=title, short title, abstract, full text, keywords, caption text] (37)

11 Speech Recognition Software.mp. [mp=title, short title, abstract, full text, keywords, caption text] (1)

12 reminder systems.mp. [mp=title, short title, abstract, full text, keywords, caption text] (37)

13 telemetry.mp. [mp=title, short title, abstract, full text, keywords, caption text] (9)

14 voice response.mp. [mp=title, short title, abstract, full text, keywords, caption text] (54)

15 1 or 2 or 3 or 4 or 5 or 6 or 7 or 8 or 9 or 10 or 11 or 12 or 13 or 14 (188)

16 heart failure.mp. [mp=title, short title, abstract, full text, keywords, caption text] (495)

17 coronary disease.mp. [mp=title, short title, abstract, full text, keywords, caption text] (99)

18 (coronary adj2 disease).mp. [mp=title, short title, abstract, full text, keywords, caption text] (383)

19 myocardial infarction.mp. [mp=title, short title, abstract, full text, keywords, caption text] (652)

20 CABG.mp. [mp=title, short title, abstract, full text, keywords, caption text] (79)

21 PCI.mp. [mp=title, short title, abstract, full text, keywords, caption text] (43)

22 PTCA.mp. [mp=title, short title, abstract, full text, keywords, caption text] (35)

23 coronary artery bypass.mp. [mp=title, short title, abstract, full text, keywords, caption text] (106)

24 angioplasty.mp. [mp=title, short title, abstract, full text, keywords, caption text] (120)

25 balloon.mp. [mp=title, short title, abstract, full text, keywords, caption text] (152)

26 coronary artery disease.mp. [mp=title, short title, abstract, full text, keywords, caption text] (173)

27 heart attack.mp. [mp=title, short title, abstract, full text, keywords, caption text] (98)

28 heart infarction.mp. [mp=title, short title, abstract, full text, keywords, caption text] (17)

29 cardiac arrest.mp. [mp=title, short title, abstract, full text, keywords, caption text] (136)

30 acute coronary syndrome.mp. [mp=title, short title, abstract, full text, keywords, caption text] (54)

31 acs.mp. [mp=title, short title, abstract, full text, keywords, caption text] (65)

32 cardiac failure.mp. [mp=title, short title, abstract, full text, keywords, caption text] (126)

33 ischemic heart disease.mp. [mp=title, short title, abstract, full text, keywords, caption text] (54)

34 angina.mp. [mp=title, short title, abstract, full text, keywords, caption text] (276)

35 16 or 17 or 18 or 19 or 20 or 21 or 22 or 23 or 24 or 25 or 26 or 27 or 28 or 29 or 30 or 31 or 32 or 33 or 34 (1352)

36 15 and 35 (61)

## Table A: Classification of telemedicine interventions

| **Intervention** | **Description** |
| --- | --- |
| Structured telephone support (STS) | STS interventions involved regular telephone contact between patients and healthcare providers to enquire about symptoms as well as provide patients with information and counselling throughout their treatment.  The delivery of the calls may be automated using interactive voice response systems where patients are expected to respond to a series of questions using the telephone keypad or from person to person using standard telephone technology. |
| Telemonitoring (TM) | TM interventions involved the use of technology to collect and transmit physiologic measurements such as weight, blood pressure, and heart rate.  This data would be monitored by healthcare providers in order to provide feedback to patients regarding their treatment and opportunities for the healthcare team to intervene as needed. |
| Structured telephone support alongside Telemonitoring | Interventions where participants received both structured telephone follow-up as well as a telemonitoring intervention that involved the transmission of physiologic and other non-invasive data. |
| Video monitoring | Video monitoring interventions involved the constituents of telemonitoring with the added function of video monitoring.  This made it possible for health professionals to provide consultation, conferencing, and/or counseling more interactively via video technology while maintaining the impact of regular physiologic monitoring of telemonitoring. |
| ECG monitoring | Alongside the constituents of telemonitoring which involved the measurement and monitoring of vital signs such as pulse, blood pressure and/or weight, clinical status was also assessed using periodic transmissions of ECG. |

**Note**: All aforementioned interventions had to be delivered periodically in a structured manner rather than on an “as needed” basis.

## WinBUGS code of the random effects model used to conduct the primary analyses.

WinBUGS Code

# Binomial likelihood, cloglog link

# Random effects model for multi-arm trials

model{ # *** PROGRAM STARTS

for(i in 1:ns){ # LOOP THROUGH STUDIES

w[i,1] <- 0 # adjustment for multi-arm trials is zero for control arm

delta[i,1] <- 0 # treatment effect is zero for control arm

mu[i] ~ dnorm(0,.0001) # vague priors for all trial baselines

for (k in 1:na[i]) { # LOOP THROUGH ARMS

r[i,k] ~ dbin(p[i,k],n[i,k]) # Binomial likelihood

cloglog(p[i,k]) <- log(time[i]) + mu[i] + delta[i,k] # model for linear predictor

rhat[i,k] <- p[i,k] * n[i,k] # expected value of the numerators

dev[i,k] <- 2 * (r[i,k] * (log(r[i,k])-log(rhat[i,k]))

+ (n[i,k]-r[i,k]) * (log(n[i,k]-r[i,k]) - log(n[i,k]-rhat[i,k]))) #Deviance contribution

}

resdev[i] <- sum(dev[i,1:na[i]]) # summed residual deviance contribution for this trial

for (k in 2:na[i]) { # LOOP THROUGH ARMS

delta[i,k] ~ dnorm(md[i,k],taud[i,k]) # trial-specific LOR distributions

md[i,k] <- d[t[i,k]] - d[t[i,1]] + sw[i,k] # mean of LOR distributions (with multi-arm correction)

taud[i,k] <- tau *2*(k-1)/k # precision of LOR distributions (with multi-arm correction)

w[i,k] <- (delta[i,k] - d[t[i,k]] + d[t[i,1]]) # adjustment for multi-arm RCTs

sw[i,k] <- sum(w[i,1:k-1])/(k-1) # cumulative adjustment for multi-arm trials

}

}

totresdev <- sum(resdev[]) #Total Residual Deviance

d[1]<-0 # treatment effect is zero for reference treatment

for (k in 2:nt){ d[k] ~ dnorm(0,.0001) } # vague priors for treatment effects

sd ~ dunif(0,5) # vague prior for between-trial SD

tau <- pow(sd,-2) # between-trial precision = (1/between-trial variance)

} # *** PROGRAM ENDS

**Note**: This code is available in the following reference:

Dias, S., Welton, N.J., Sutton, A.J. & Ades, A.E. NICE DSU Technical Support Document 2: A Generalised Linear Modelling Framework for Pairwise and Network Meta-Analysis of Randomised Controlled Trials. 2011; last updated April 2014; available from http://www.nicedsu.org.uk

## Table B: Characteristics of included reviews

| **Author/Year** | **Included studies** | **Patients** | **Intervention/**  **Comparison** | **Outcomes** |
| --- | --- | --- | --- | --- |
| Chaudhry 2007 | 9 Randomized controlled studies | Adult heart failure patients only | I: regular nurse delivered telephone calls over 6-12+ months to monitor symptoms  I: twice daily monitoring of symptoms and weight for 6 months  I: for 3 months daily monitoring of weight blood pressure HR and oxygen saturation compared with home nurse visits  I: physiologic monitoring consisting of twice daily self-measurements and monthly calls by nurse to assess symptoms and meds  I: video conferencing with integrated stethoscope and nurse telephone support  C: usual care | All-cause hospitalization  HF-hospitalization  Mortality |
| Clark 2007 | 14 Randomized controlled studies | Chronic HF patients at home | Structured telephone support: included monitoring of symptoms medicine management and education and counseling on life-style.  Telemonitoring: included transfer of daily data on weight pulse blood pressure and electrocardiographic findings. | All-cause hospitalization  HF-hospitalization  Mortality |
| Clarke 2011 | 13 Randomized controlled studies | Congestive HF at home | Telemonitoring (no telephone only intervention; Collected data on signs, symptoms and physiologic measurement | All-cause hospitalization  HF-hospitalization  Mortality |
| Dang 2009 | 9 Randomized controlled studies | Congestive HF | Automated monitoring or automated physiologic monitoring; excluded telephone-only monitoring interventions | All-cause hospitalization  HF-hospitalization  Mortality |
| Giamouzis 2012 | 12 Randomized controlled studies | Chronic HF | Telemonitoring measuring physiological parameter vs. usual care | All-cause hospitalization  Mortality |
| Holland 2004 | 3 Randomized controlled studies | Congestive HF | Videophone or any form of home physiological monitoring (group B) vs. usual care | All-cause hospitalization  HF-hospitalization  Mortality |
| Inglis 2011 [including abstracts Clark S100; Clark 944-945; Inglis S179; Inglis 1028-1040; Inglis S90; Inglis 878] | 29 Randomized controlled studies | Chronic HF | TM and Structured TF delivered by health professionals for discharged patients who are not also getting intensified follow up or home visits vs. usual care | All-cause hospitalization  HF-hospitalization  Mortality |
| Klersy 2011 | 21 Randomized controlled studies | HF | Remote patient monitoring (regular structured telephone or electronic transfer of physiological data) vs. usual care | All-cause hospitalization |

###

## Table C: Assessing the quality of included reviews

| **Study** | **AMSTAR Criteria** | | | | | | | | | | |
| --- | --- | --- | --- | --- | --- | --- | --- | --- | --- | --- | --- |
|  | **(1)** | **(2)** | **(3)** | **(4)** | **(5)** | **(6)** | **(7)** | **(8)** | **(9)** | **(10)** | **(11)** |
| Chaudhry 2007 | Can’t answer | Yes | Yes | No | No | Yes | Yes | Yes | NA | No | Yes |
| Clark 2007 | Can’t answer | Yes | Yes | Yes | No | Yes | Yes | No | Yes | No | No |
| Clarke 2011 | Can’t answer | Yes | Yes | No | No | Yes | No | No | No | No | No |
| Dang 2009 | Can’t answer | Yes | Yes | No | Yes | Yes | Yes | Yes | NA | No | Yes |
| Giamouzis 2012 | Can’t answer | Yes | Yes | No | No | Yes | No | No | NA | No | Yes |
| Holland 2013 | Can’t answer | Yes | Yes | No | No | Yes | Yes | Yes | Yes | No | No |
| Inglis 2011 | Can’t answer | Yes | Yes | Yes | Yes | Yes | Yes | Yes | No | Yes | Yes |
| Klersy 2009 (and Klersy 2010) | Can’t answer | Yes | Yes | No | Yes | Yes | Yes | Yes | Yes | Yes | No |

**Summary of the AMSTAR Assessment. Note:** *NA=Not applicable.* *(1) Was an 'a priori' design provided? (2) Was there duplicate study selection and data extraction? (3) Was a comprehensive literature search performed? (4) Was the status of publication (i.e. grey literature) used as an inclusion criterion? (5) Was a list of studies (included and excluded) provided? (6) Were the characteristics of the included studies provided? (7) Was the scientific quality of the included studies assessed and documented? (8) Was the scientific quality of the included studies used appropriately in formulating conclusions? (9) Were the methods used to combine the findings of studies appropriate? (10) Was the likelihood of publication bias assessed? (11) Was the conflict of interest included?*

## Table D: Quality assessment of included trials

| **Study** | **SIGN-50** | | | | | | | | | | |
| --- | --- | --- | --- | --- | --- | --- | --- | --- | --- | --- | --- |
|  | **1.1** | **1.2** | **1.3** | **1.4** | **1.5** | **1.6** | **1.7** | **1.8** | **1.9** | **1.10** | **2.1** |
| Balk 2008 | Yes | Yes | Yes | No | Yes | Yes | Yes | Can’t say | Yes | Yes | ++ |
| Blum 2007 | Yes | Can’t say | Can’t say | No | Can’t say | Can’t say | Can’t say | Can’t say | Can’t say | Can’t say | + |
| Capomolla 2004 | Yes | Yes | Can’t say | No | Yes | Yes | Yes | 82% | Yes | No | ++ |
| Cleland 2005 | Yes | Yes | Yes | No | Can’t say | Can’t say | Yes | >20% | Yes | Can’t say | ++ |
| De Lusignan 2001 | Yes | Yes | Can’t say | No | Can’t say | Can’t say | Yes | 20% | Can’t say | NA | 0 |
| DeBusk 2004 | Yes | Yes | Yes | No | Yes | Yes | Yes | <20% | Yes | NA | ++ |
| Dendale 2012 | Yes | Yes | Yes | No | Yes | Yes | Yes | Can’t say | Yes | NA | ++ |
| DeWalt 2006 | Yes | Yes | Yes | No | Yes | Yes | Yes | <20% | Can’t say | NA | ++ |
| Ekman 1998 | Yes | Yes | Yes | No | Yes | No | Yes | <20% | Yes | NA | ++ |
| Galbreath 2004 | Yes | Yes | Can’t say | No | Yes | No | Yes | Can’t say | Yes | NA | ++ |
| GESICA 2005 | Yes | Yes | Yes | No | Yes | Yes | Yes | <20% | Yes | Can’t say | ++ |
| Giordano 2009 | Yes | Yes | Can’t say | No | Yes | No | Yes | <20% | Yes | Can’t say | ++ |
| Goldberg 2003 | Yes | Yes | Can’t say | No | Yes | Yes | Yes | Can’t say | Yes | Can’t say | ++ |
| Kielblock 2007 | Yes | Yes | Can’t say | No | No | No | Yes | Can’t say | Can’t say | Can’t say | 0 |
| Koehler 2011 | Yes | Yes | Can’t say | No | Yes | Yes | Yes | >20% | Yes | Can’t say | ++ |
| Krum 2009 | Yes | Yes | Can’t say | No | Yes | Yes | Yes | <20% | Can’t say | Can’t say | + |
| Krumholz 2002 | Yes | Yes | Can’t say | No | Yes | No | Yes | Can’t say | Yes | NA | + |
| Laramee 2003 | Yes | Yes | Can’t say | No | Yes | No | Yes | >20% | Yes | NA | + |
| Mortara 2009 | Yes | Yes | Yes | No | Yes | No | Yes | Can’t say | Can’t say | Can’t say | + |
| Ramachandran 2007 | Yes | Yes | Can’t say | No | Yes | No | Yes | <20% | Can’t say | NA | + |
| Riegel 2002 | Yes | Yes | Can’t say | No | Yes | No | Yes | Can’t say | Can’t say | NA | 0 |
| Riegel 2006 | Yes | Yes | Yes | No | Yes | Yes | Yes | <20% | Yes | NA | ++ |
| Schwarz 2008 | Yes | Yes | Yes | No | Yes | No | Yes | >20% | Yes | NA | ++ |
| Sisk 2006 | Yes | Yes | Yes | No | Yes | Yes | Yes | >20% | Yes | NA | ++ |
| Villani 2007 | Yes | Yes | Can’t say | No | Yes | No | Yes | Can’t say | Can’t say | NA | + |
| Wade 2011 | Yes | Yes | Can’t say | No | Yes | No | Yes | >20% | Yes | NA | + |
| Wakefield 2008 | Yes | Yes | Yes | No | Yes | Yes | Yes | >20% | Yes | NA | ++ |
| Woodend 2008 | Yes | Yes | Can’t say | No | Yes | No | Yes | <20% | Yes | NA | ++ |
| Zugck 2005 | Yes | Yes | Can’t say | No | Can’t say | Can’t say | Yes | Can’t say | Can’t say | NA | + |

**Summary of the SIGN-50 assessment. Note:** *NA=Not applicable. High quality (++) Acceptable (+) Unacceptable (0). 1.1 The study addresses an appropriate and clearly focused question. 1.2 The assignment of subjects to treatment groups is randomized. 1.3 An adequate concealment method is used. 1.4 Subjects and investigators are kept ‘blind’ about treatment allocation. 1.5 The treatment and control groups are similar at the start of the trial. 1.6 The only difference between groups is the treatment under investigation. 1.7 All relevant outcomes are measured in a standard, valid and reliable way. 1.8 What percentage of the individuals or clusters recruited into each treatment arm of the study dropped out before the study was completed? 1.9 All the subjects are analysed in the groups to which they were randomly allocated (often referred to as intention to treat analysis). 1.10 Where the study is carried out at more than one site, results are comparable for all sites. 2.1 How well was the study done to minimise bias?*

**
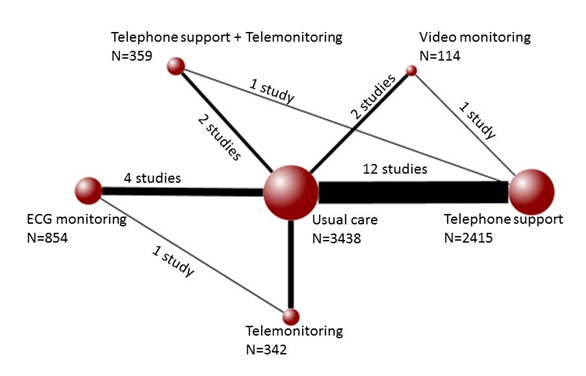
 Figure A: Evidence networks for all-cause hospitalization and heart failure hospitalization. Evidence networks for interventions included in the analysis of all-cause hospitalization.**

**
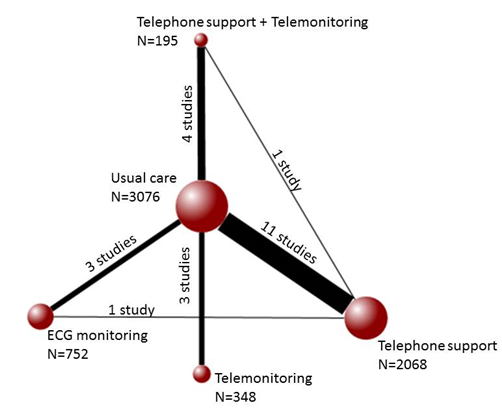
**

**Figure B: Evidence networks for heart failure hospitalization. Evidence networks for interventions included in the analysis of heart failure hospitalization.**

**
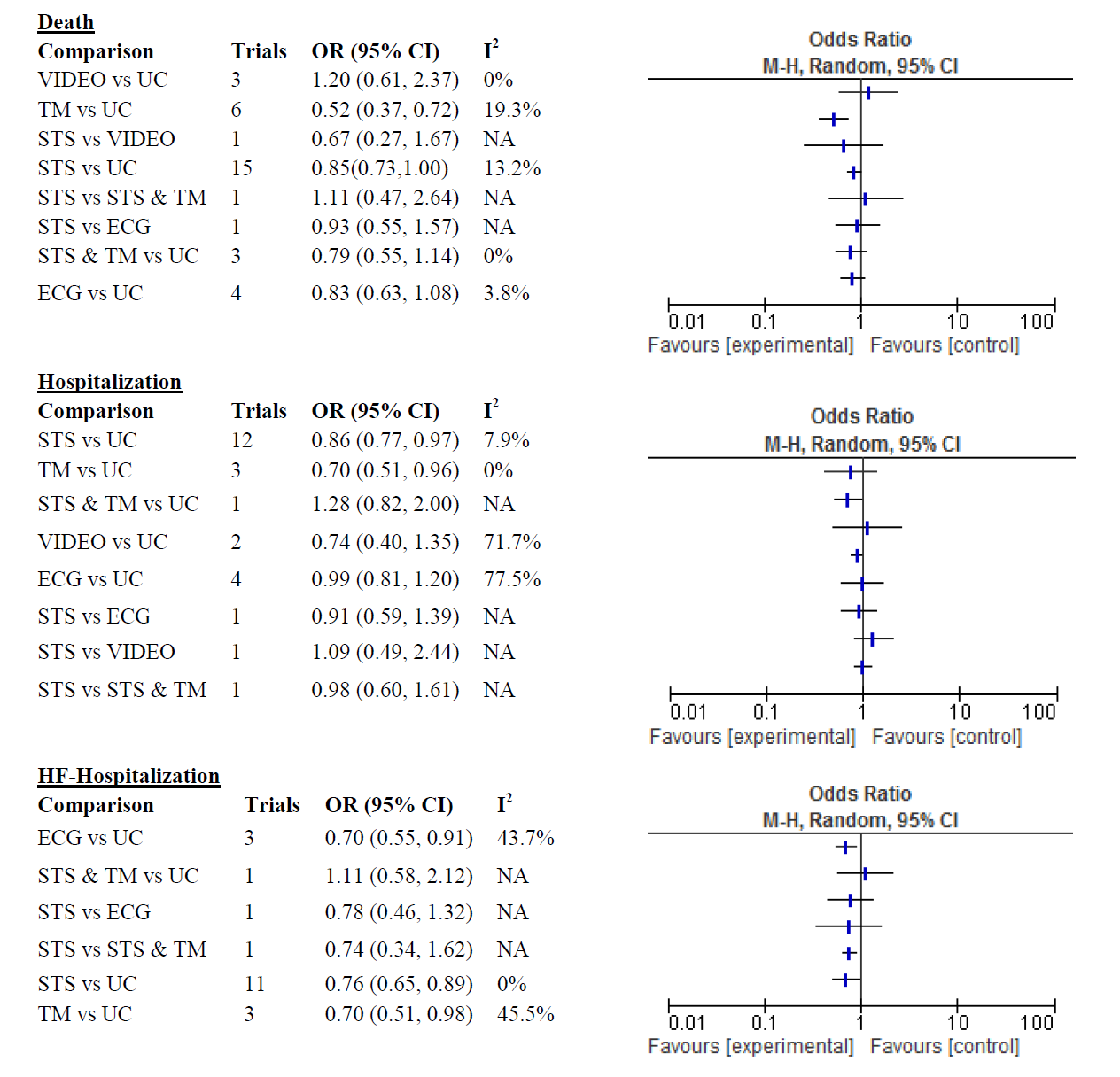
**

**Figure C: Summary of the direct pairwise comparisons. Estimates of odds ratios for pairwise comparisons between usual care (UC) structured telephone support (STS), telemedicine, video monitoring (Video) and telemedicine involving ECG (ECG).**

## Comparison across models for each outcome

For the analysis of all outcomes, the consistency models had lower DIC values than the inconsistency models and residual deviances were comparable to the number of unconstrained data points. This indicated the goodness of fit of the selected models. Compared to the fixed effects models, the random effects models had lower residual deviance values and were therefore used to analyze all outcomes.

**Table E: DIC and Residual Deviances across different models for each outcome**

| **Outcome: death** | **Random Effects Model** | **Fixed Effects Model** | **Inconsistency Model** |
| --- | --- | --- | --- |
| ***Datapoints*** | 61 | 61 | 61 |
| ***Residual deviance*** | 56.96 | 60.74 | 58.54 |
| ***DIC*** | 358.341 | 357.422 | 360.763 |
| **Outcome: hospitalization** | **Random Effects Model** | **Fixed Effects Model** | **Inconsistency Model** |
| ***Datapoints*** | 43 | 43 | 43 |
| ***Residual deviance*** | 45.84 | 55.61 | 46.41 |
| ***DIC*** | 302.364 | 304.619 | 303.922 |
| **Outcome: Heart failure hospitalization** | **Random Effects Model** | **Fixed Effects Model** | **Inconsistency Model** |
| ***Datapoints*** | 34 | 34 | 34 |
| ***Residual deviance*** | **33.32** | 35.41 | 33.36 |
| ***DIC*** | 223.202 | 221.678 | 223.096 |

## Inconsistency plots

Inconsistency was examined by plotting the deviances from the consistency model against the deviances from the inconsistency model for each outcome. There was little evidence of inconsistency from the plots.

**
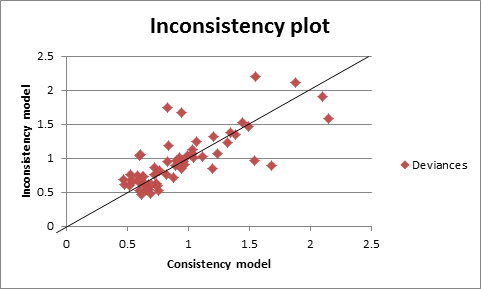
**

**Figure D: Inconsistency plot for the outcome of Death**

**
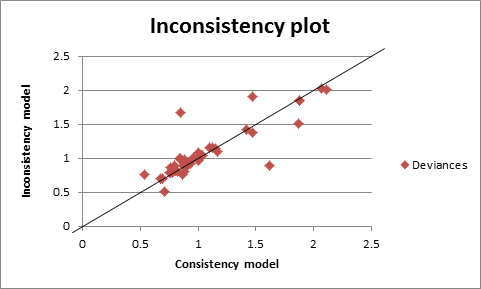
**

**Figure E: Inconsistency plot for the outcome of Hospitalization**

**
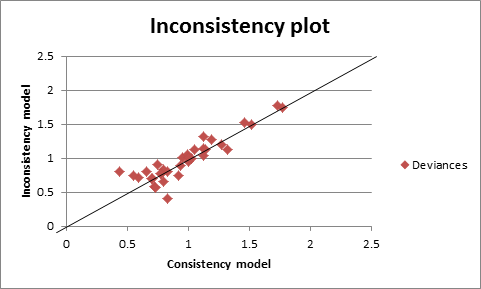
**

**Figure F: Inconsistency plot for the outcome of Hospitalization due to heart failure**

## Comparing between models

The effect estimates were also compared between models for treatment comparisons having direct evidence and were found to be similar across both models.

**Table F: Comparing between models for the outcome of Death**

| **node** | **OR-Consistency model** | | | **OR-Inconsistency model** | | |
| --- | --- | --- | --- | --- | --- | --- |
|  | **2.50%** | **median** | **97.50%** | **2.50%** | **median** | **97.50%** |
| **or[Usual Care, Structured telephone support]** | 0.6589 | 0.7972 | 0.9603 | 0.6559 | 0.7983 | 0.9605 |
| **or[Usual Care, Telemonitoring]** | 0.3634 | 0.5312 | 0.7969 | 0.3542 | 0.5269 | 0.7978 |
| **or[Usual Care, Structured telephone support plus Telemonitoring]** | 0.5082 | 0.7664 | 1.154 | 0.5033 | 0.7721 | 1.2057 |
| **or[Usual Care, Video monitoring]** | 0.5845 | 1.176 | 2.345 | 0.5736 | 1.1716 | 2.3843 |
| **or[Usual Care, ECG monitoring]** | 0.5665 | 0.7838 | 1.064 | 0.5592 | 0.7837 | 1.0710 |
| **or[Structured telephone support, Structured telephone support plus Telemonitoring]** | 0.6262 | 0.9615 | 1.484 | 0.2585 | 0.9035 | 3.4764 |

**Table G: Comparing between models for the outcome of Hospitalization**

| **Node** | **OR-Consistency model** | | | **OR-Inconsistency model** | | |
| --- | --- | --- | --- | --- | --- | --- |
|  | **2.50%** | **median** | **97.50%** | **2.50%** | **median** | **97.50%** |
| **or[Usual Care, Structured telephone support]** | 0.7394 | 0.8796 | 1.063 | 0.7285 | 0.8756 | 1.0641 |
| **or[Usual Care, Telemonitoring]** | 0.4781 | 0.7471 | 1.165 | 0.4781 | 0.7507 | 1.1963 |
| **or[Usual Care, Structured telephone support plus Telemonitoring]** | 0.6818 | 1.052 | 1.626 | 0.6313 | 1.1500 | 2.2291 |
| **or[Usual Care, Video monitoring]** | 0.3946 | 0.7981 | 1.645 | 0.3680 | 0.7845 | 1.6706 |
| **or[Usual Care, ECG monitoring]** | 0.7419 | 0.9883 | 1.337 | 0.7308 | 0.9869 | 1.3685 |
| **or[Structured telephone support, Structured telephone support plus Telemonitoring]** | 0.7816 | 1.196 | 1.81 | 0.5782 | 1.1301 | 2.1769 |

**Table H: Comparing between models for the outcome of HF-Hospitalization**

| **Node** | **OR-Consistency model** | | | **OR-Inconsistency model** | | |
| --- | --- | --- | --- | --- | --- | --- |
|  | **2.50%** | **median** | **97.50%** | **2.50%** | **median** | **97.50%** |
| **or[Usual Care, Structured telephone support]** | 0.5621 | 0.6911 | 0.8462 | 0.5626 | 0.6911 | 0.8503 |
| **or[Usual Care, Telemonitoring]** | 0.3932 | 0.6374 | 0.9479 | 0.3937 | 0.6364 | 0.9502 |
| **or[Usual Care, Structured telephone support plus Telemonitoring]** | 0.5256 | 1.034 | 1.99 | 0.5280 | 1.0437 | 2.1327 |
| **or[Usual Care, ECG monitoring]** | 0.5161 | 0.7083 | 0.9805 | 0.5013 | 0.7055 | 0.9775 |

## Sensitivity analysis results

Studies were considered to be of high quality if they satisfied all of the aforementioned criteria. When trials that were judged to have a high risk of bias were excluded, the number of studies included in the analysis of death, hospitalization and hospitalization due to heart failure were reduced to 26, 16, and 14, respectively. Generally, the estimates for all outcomes were consistent with the main analyses. There was one exception in that telemonitoring was no longer found to significantly reduce deaths compared to usual care [OR 0.64 95% CrI (0.36, 1.12)].

**Table I: Sensitivity analysis excluding studies with low quality, according to SIGN-50, for the outcome of death.**

| **Primary analysis** | **OR [Credible intervals]** | | | **Sign 50 (-) excluded** | **OR [Credible intervals]** | | |
| --- | --- | --- | --- | --- | --- | --- | --- |
|  | **2.50%** | **median** | **97.50%** |  | **2.50%** | **median** | **97.50%** |
| or[UC,STS] | 0.6589 | 0.7972 | 0.9603 | or[UC,STS] | 0.6475 | 0.7937 | 0.9672 |
| **or[UC,TM]** | **0.3634** | **0.5312** | **0.7969** | **or[UC,TM]** | **0.3584** | **0.635** | **1.121** |
| or[UC,RPM] | 0.5082 | 0.7664 | 1.154 | or[UC,RPM] | 0.5031 | 0.7579 | 1.162 |
| or[UC,Video] | 0.5845 | 1.176 | 2.345 | or[UC,Video] | 0.6077 | 1.281 | 2.647 |
| or[UC,ECG] | 0.5665 | 0.7838 | 1.064 | or[UC,ECG] | 0.5592 | 0.7818 | 1.068 |
| or[STS,TM] | 0.4361 | 0.6669 | 1.047 | or[STS,TM] | 0.4324 | 0.8028 | 1.467 |
| or[STS,RPM] | 0.6262 | 0.9615 | 1.484 | or[STS,RPM] | 0.62 | 0.9573 | 1.497 |
| or[STS,Video] | 0.7196 | 1.476 | 2.975 | or[STS,Video] | 0.7521 | 1.617 | 3.361 |
| or[STS,ECG] | 0.6878 | 0.9827 | 1.385 | or[STS,ECG] | 0.6812 | 0.9855 | 1.401 |
| or[TM,RPM] | 0.8142 | 1.434 | 2.546 | or[TM,RPM] | 0.5827 | 1.197 | 2.442 |
| or[TM,Video] | 0.994 | 2.212 | 4.929 | or[TM,Video] | 0.7817 | 2.013 | 5.11 |
| or[TM,ECG] | 0.8717 | 1.476 | 2.394 | or[TM,ECG] | 0.6287 | 1.229 | 2.359 |
| or[RPM,Video] | 0.6847 | 1.542 | 3.445 | or[RPM,Video] | 0.7193 | 1.687 | 3.854 |
| or[RPM,ECG] | 0.6069 | 1.021 | 1.703 | or[RPM,ECG] | 0.5992 | 1.027 | 1.714 |
| or[Video,ECG] | 0.3144 | 0.6672 | 1.42 | or[Video,ECG] | 0.2739 | 0.6087 | 1.359 |

**Note:** STS = structured telephone support. TM = telemonitoring. RPM = structured telephone support alongside telemonitoring. Video = video monitoring. ECG = telemedicine using electrocardiography. The main difference observed is in bold and underlined.

**Table J: Sensitivity analysis excluding studies with low quality, according to SIGN-50, for the outcome of hospitalization.**

| **Primary analysis** | **OR [Credible intervals]** | | | **Sign 50 (-) excluded** | **OR [Credible intervals]** | | |
| --- | --- | --- | --- | --- | --- | --- | --- |
|  | **2.50%** | **median** | **97.50%** |  | **2.50%** | **median** | **97.50%** |
| or[UC,STS] | 0.7394 | 0.8796 | 1.063 | or[UC,STS] | 0.7345 | 0.8962 | 1.108 |
| or[UC,TM] | 0.4781 | 0.7471 | 1.165 | or[UC,TM] | 0.3779 | 0.8117 | 1.733 |
| or[UC,RPM] | 0.6818 | 1.052 | 1.626 | or[UC,RPM] | 0.6645 | 1.063 | 1.707 |
| or[UC,Video] | 0.3946 | 0.7981 | 1.645 | or[UC,Video] | 0.3982 | 0.8141 | 1.725 |
| or[UC,ECG] | 0.7419 | 0.9883 | 1.337 | or[UC,ECG] | 0.7262 | 0.9969 | 1.389 |
| or[STS,TM] | 0.5232 | 0.8489 | 1.362 | or[STS,TM] | 0.4101 | 0.9049 | 1.975 |
| or[STS,RPM] | 0.7816 | 1.196 | 1.81 | or[STS,RPM] | 0.7466 | 1.186 | 1.867 |
| or[STS,Video] | 0.4454 | 0.9071 | 1.872 | or[STS,Video] | 0.4404 | 0.907 | 1.917 |
| or[STS,ECG] | 0.8098 | 1.123 | 1.565 | or[STS,ECG] | 0.7741 | 1.113 | 1.601 |
| or[TM,RPM] | 0.7528 | 1.409 | 2.62 | or[TM,RPM] | 0.5402 | 1.313 | 3.2 |
| or[TM,Video] | 0.4719 | 1.064 | 2.525 | or[TM,Video] | 0.3549 | 1.005 | 2.927 |
| or[TM,ECG] | 0.7856 | 1.321 | 2.267 | or[TM,ECG] | 0.5446 | 1.228 | 2.809 |
| or[RPM,Video] | 0.3378 | 0.7574 | 1.758 | or[RPM,Video] | 0.3328 | 0.7644 | 1.832 |
| or[RPM,ECG] | 0.5624 | 0.9399 | 1.581 | or[RPM,ECG] | 0.5355 | 0.9399 | 1.658 |
| or[Video,ECG] | 0.5671 | 1.239 | 2.639 | or[Video,ECG] | 0.5479 | 1.224 | 2.675 |

**Note**: STS = structured telephone support. TM = telemonitoring. RPM = structured telephone support alongside telemonitoring. Video = video monitoring. ECG = telemedicine using electrocardiography.

**Table K: Sensitivity analysis excluding studies with low quality, according to SIGN-50, for the outcome of heart failure hospitalization.**

| **Primary analysis** | **OR [Credible intervals]** | | | **Sign 50 (-) excluded** | **OR [Credible intervals]** | | |
| --- | --- | --- | --- | --- | --- | --- | --- |
|  | **2.50%** | **median** | **97.50%** |  | **2.50%** | **median** | **97.50%** |
| or[UC,STS] | 0.5621 | 0.6911 | 0.8462 | or[UC,STS] | 0.5683 | 0.7065 | 0.8708 |
| or[UC,TM] | 0.3932 | 0.6374 | 0.9479 | or[UC,TM] | 0.1732 | 0.365 | 0.7511 |
| or[UC,RPM] | 0.5256 | 1.034 | 1.99 | or[UC,RPM] | 0.5374 | 1.051 | 2.06 |
| or[UC,ECG] | 0.5161 | 0.7083 | 0.9805 | or[UC,ECG] | 0.518 | 0.7137 | 0.9758 |
| or[STS,TM] | 0.545 | 0.9238 | 1.426 | or[STS,TM] | 0.2403 | 0.5196 | 1.091 |
| or[STS,RPM] | 0.7466 | 1.497 | 2.922 | or[STS,RPM] | 0.7559 | 1.495 | 2.96 |
| or[STS,ECG] | 0.7155 | 1.026 | 1.481 | or[STS,ECG] | 0.7055 | 1.012 | 1.442 |
| or[TM,RPM] | 0.7389 | 1.622 | 3.686 | or[TM,RPM] | 1.068 | 2.868 | 7.806 |
| or[TM,ECG] | 0.6818 | 1.11 | 2.009 | or[TM,ECG] | 0.896 | 1.949 | 4.375 |
| or[RPM,ECG] | 0.3323 | 0.6835 | 1.469 | or[RPM,ECG] | 0.3243 | 0.6795 | 1.418 |

**Note:** STS = structured telephone support. TM = telemonitoring. RPM = structured telephone support alongside telemonitoring. ECG = telemedicine using electrocardiography.

## Network meta-analysis results of fixed effects model

For all comparisons and across all outcomes, the results of both the fixed-effect and the random-effect model were consistent. Only on two occasions, the fixed-effect model showed a significant reduction where the random-effect model did not. According to the fixed-effect model, telemonitoring was associated with significantly lower odds of death compared to the structured telephone support intervention [OR 0.65 95% CrI (0.44, 0.94)]. This reduction was not considered significant when a random-effect model was used [OR 0.67 95% CrI [0.44, 1.06)]. The same situation occurred when structured telephone support was compared to usual care for the outcome of all-cause hospitalization. The fixed-effect model estimate was found to be significant [OR 0.86 95% CrI (0.77, 0.97)] whereas the estimate from the random-effect model was not [OR 0.88 95% CrI (0.74, 1.06)]. Estimates from the fixed effects models are listed below.

**Table L: Direct and indirect comparisons for death**

| **Comparison** | **Odds ratios (97.5% Credible intervals)** |
| --- | --- |
| Usual care vs. structured telephone support | 0.81 (0.69, 0.94) |
| Usual care vs. Telemonitoring | 0.52 (0.37, 0.73) |
| Usual care vs. structured telephone support and Telemonitoring | 0.75 (0.53, 1.01) |
| Usual care vs. telemedicine involving video monitoring | 1.19 (0.62, 2.28) |
| Usual care vs. telemedicine involving electrocardiographic data transmission | 0.80 (0.62, 1.03) |
| Structured telephone support vs. Telemonitoring | 0.65 (0.44, 0.94) |
| Structured telephone support vs. structured telephone support and Telemonitoring | 0.94 (0.65, 1.35) |
| Structured telephone support vs. telemedicine involving video monitoring | 1.47 (0.77, 2.84) |
| Structured telephone support vs. telemedicine involving electrocardiographic data transmission | 0.99 (0.75, 1.31) |
| Telemonitoring vs. structured telephone support and Telemonitoring | 1.45 (0.89, 2.35) |
| Telemonitoring vs. telemedicine involving video monitoring | 2.28 (1.09, 4.77) |
| Telemonitoring vs. telemedicine involving electrocardiographic data transmission | 1.54 (1.005, 2.35) |
| Structured telephone support and Telemonitoring vs. video monitoring | 1.58 (0.76, 3.28) |
| Structured telephone support and Telemonitoring vs. telemedicine involving electrocardiographic data transmission | 1.06 (0.69, 1.63) |
| Video monitoring vs. telemedicine involving electrocardiographic data transmission | 0.67 (0.34, 1.35) |

**Table M: Direct and indirect comparisons for hospitalization**

| **Comparison** | **Odds ratios (97.5% Credible intervals)** |
| --- | --- |
| Usual care vs. structured telephone support | 0.86 (0.77, 0.97) |
| Usual care vs. Telemonitoring | 0.73 (0.53, 1.01) |
| Usual care vs. structured telephone support and Telemonitoring | 1.05 (0.77, 1.43) |
| Usual care vs. telemedicine involving video monitoring | 0.77 (0.41, 1.44) |
| Usual care vs. telemedicine involving electrocardiographic data transmission | 0.98 (0.81, 1.19) |
| Structured telephone support vs. Telemonitoring | 0.85 (0.60, 1.20) |
| Structured telephone support vs. structured telephone support and Telemonitoring | 1.21 (0.89, 1.64) |
| Structured telephone support vs. telemedicine involving video monitoring | 0.89 (0.47, 1.67) |
| Structured telephone support vs. telemedicine involving electrocardiographic data transmission | 1.13 (0.92, 1.41) |
| Telemonitoring vs. structured telephone support and telemedicine | 1.42 (0.91, 2.23) |
| Telemonitoring vs. telemedicine involving video monitoring | 1.04 (0.51, 2.12) |
| Telemonitoring vs. telemedicine involving electrocardiographic data transmission | 1.34 (0.92, 1.94) |
| Structured telephone support and Telemonitoring vs. telemedicine involving video monitoring | 0.73 (0.37, 1.47) |
| Structured telephone support and Telemonitoring vs. telemedicine involving electrocardiographic data transmission | 0.94 (0.65, 1.35) |
| Telemedicine involving Video monitoring vs. telemedicine involving electrocardiographic data transmission | 1.28 (0.66 2.47) |

**Table N: Direct and indirect comparisons for HF-hospitalization**

| **Comparison** | **Odds ratios (97.5% Credible intervals)** |
| --- | --- |
| Usual care vs. structured telephone support | 0.69 (0.59, 0.81) |
| Usual care vs. Telemonitoring | 0.67 (0.48, 0.94) |
| Usual care vs. structured telephone support and Telemonitoring | 1.04 (0.58, 1.87) |
| Usual care vs. telemedicine involving electrocardiographic data transmission | 0.71 (0.56, 0.91) |
| Structured telephone support vs. Telemonitoring | 0.97 (0.67, 1.41) |
| Structured telephone support vs. structured telephone support and Telemonitoring | 1.51 (0.83, 2.72) |
| Structured telephone support vs. telemedicine involving electrocardiographic data transmission | 1.03 (0.78, 1.35) |
| Telemonitoring vs. structured telephone support and Telemonitoring | 1.55 (0.79, 3.03) |
| Telemedicine vs. telemedicine involving electrocardiographic data transmission | 1.06 (0.70, 1.60) |
| Structured telephone support and Telemonitoring vs. telemedicine involving electrocardiographic data transmission | 0.68 (0.36, 1.29) |
